# Modeling and predicting meat yield and growth performance using morphological features of narrow-clawed crayfish with machine learning techniques

**DOI:** 10.1038/s41598-024-69539-5

**Published:** 2024-08-09

**Authors:** Yasemin Gültepe, Selçuk Berber, Nejdet Gültepe

**Affiliations:** 1 Faculty of Engineering, Department of Software Engineering, Atatürk University, 25240 Erzurum, Türkiye; 2 Faculty of Marine Science and Technology, Department of Fisheries Fundamental Sciences, Çanakkale Onsekiz Mart University, 17100 Çanakkale, Türkiye; 3 Faculty of Fisheries, Department of Fisheries Fundamental Sciences, Atatürk University, 25240 Erzurum, Türkiye

**Keywords:** Support vector regression, Artificial neural network, Machine learning, *Pontastacus leptodactylus*, Crayfish, Sustainable fisheries, Ecological modelling, Limnology, Freshwater ecology

## Abstract

In recent studies, artificial intelligence and machine learning methods give higher accuracy than other prediction methods in large data sets with complex structures. Instead of statistical methods, artificial intelligence, and machine learning are used due to the difficulty of constructing mathematical models in multi-parameter and multivariate problems. In this study, predictions of length–weight relationships and meat productivity were generated by machine learning models using measurement data of male and female crayfish in the narrow-clawed crayfish population living in Apolyont Lake. The data set was created using the growth performance and morphometric characters from 1416 crayfish in different years to determine the length–weight relationship and length-meat yield. Statistical methods, artificial intelligence, and machine learning are used due to the difficulty of constructing mathematical models in multi-parameter and multivariate problems. The analysis results show that most models designed as an alternative to traditional estimation methods in future planning studies in sustainable fisheries, aquaculture, and natural sources management are valid for machine learning and artificial intelligence. Seven different machine learning algorithms were applied to the data set and the length–weight relationships and length-meat yields were evaluated for both male and female individuals. Support vector regression (SVR) has achieved the best prediction performance accuracy with 0.996 and 0.992 values for the length–weight of males and females, with 0.996 and 0.995 values for the length-meat yield of males and females. The results showed that the SVR outperforms the others for all scenarios regarding the accuracy, sensitivity, and specificity metrics.

## Introduction

Freshwater lobsters, also known as crayfish, which are one of the largest inland forms of decapod crustaceans, that contain economically important species, are represented by 737 species and subspecies in the world^1,2^. The present species in Türkiye is genetically defined as a *Pontastacus leptodactylus* and has different subspecies^2–4^. Crayfish production is done through catching and breeding in the world. Despite a large number of species, catching and breeding activities generally focused on the species of only three families (Cambaridae, Parastacidae, Astacidae) that are economically important. The amount of crayfish production with catching was determined as 15,426 tons, excluding China, as of 2015, and Armenia ranks first with a production of 7380 tons. Considering the production amounts based on species, the most produced crayfish by catching is *Pontastacus leptodactylus*. Through aquaculture, 787,373 tons of crayfish have been produced in the world. China ranks first with 723,200 tons of production. *Procambarus clarkii* is the leading species produced by aquaculture with 786,905 tons^5^. In recent years, crayfish plague, overfishing, and increased water pollution caused fluctuations in crayfish production. Stock management and alternative production methods should be improved and developed due to fluctuating trends in volumes of crayfish production and the available data should be used carefully and successfully. In other words, crayfish producers must give data for processing with new technologies in this way could help decision-makers determine the right decisions and strategies for the future. However, it is not possible to manually process and analyze very large amounts of data. Many different ways in machine learning involve patterns in which relationships can be established; these determine the technique that can be used to make sense of the output from the data. The most commonly used machine learning methods in the literature are; artificial neural networks^6–8^, logistic regression^8,9^, fuzzy modeling^10^, genetic algorithms and programming^11^, decision tree^7,9^, Bayesian network approach^12–14^, random forest^9,15^, support vector machine^9,16^. Regression-based modeling techniques are widely used to estimate species distribution and water quality^17^ such as generalized additive models (GAMs), generalized linear models (GLM), classification and regression trees (CART), and multivariate adaptive regression splines (MARS). Also, a modeling approach that integrates a functional network approach with a dynamic Bayesian network model was used to predict trends of different fish and zooplankton species from specific fishing, temperature, and net primary production (Net PP) scenarios^18^. Similarly, Hamilton et al.^13^, a Bayesian network approach to developing a habitat suitability model, and Lin et al.^12^ used a Bayesian analysis to account for the combined uncertainty and variability of parameters in the crayfish bioaccumulation model. Some of the growth parameters of Tigris loach (*Oxynoemacheilus tigris*) were estimated by using both length–weight relationship and artificial neural network (ANN) between 2014 and 2015 from 14 different stations in Karasu and the two methods were compared with each other. It has been observed that there is a high affinity between the measured and predicted data and the values obtained with ANN are closer to the real values^19^. Also, Ozcan^20^ pointed out that ANN can be used as an alternative method for the estimation of population.

The study aims to reveal and predict the future status of the population by determining the meat yield and growth performance of narrow-clawed crayfish living in Lake Apolyont in different years for sustainable aquaculture and fishing. One of the goals of this study is to contribute to future research in Apolyont Lake, which is in an important market like Europe. In this context, this study consists of two modules. The first is to apply data preprocessing techniques to all data sets. In the second, random forest regression (RFR), gradient boosting regression (GBR), decision tree regression (DTR), multilayer perceptron regression (MLPR), support vector regression (SVR), linear regression (LR) and K-nearest neighbors regression (K-NNR) machine learning methods are executed all together on the dataset. For this, various machine learning algorithms were applied to the data set, and the best estimation performance of the total length–weight and meat yield of narrow-clawed crayfish was achieved.

## Materials and methods

### Study area and data collection

**Figure 1:**
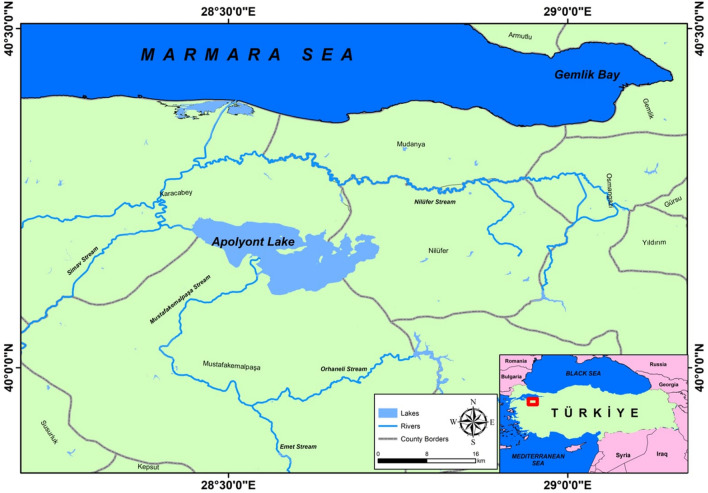
Apolyont Lake.

The dataset used in this study is created by using narrow-clawed crayfish data obtained from Apolyont Lake in Balıkesir-Türkiye. Map of the study area generated using ArcGIS Desktop version 10.8 (https://www.esri.com/en-us/arcgis/products/arcgis-desktop/overview) (Fig. 1). An individual of 1461 narrow-clawed crayfish, consisting of 573 (40%) females and 843 (60%) males, was caught. Each sample has 22 attributes, and these attributes are presented in Table 1. Length measurements of body parts of crayfish are used to determine morphological differences between male and female crayfish among species^15,16,18^. These measurements are used to determine the comparative growth of populations, the size of crayfish to be put on the market, meat yield, and systematic separation. Length measurements of narrow-clawed crayfish broodstock and juvenile individuals were made with a digital caliper with 0.01 mm precision. Length measurements are based on the total length from the rostrum point to the telson point. In the measurement of the weight of narrow-clawed crayfish, the weight of the broodstock was measured with a 0.01 g precision weighing, while the weight of the juvenile individuals was measured with a 0.0001 g precision scale^21^.

**Table 1:** Attribute information.

Number	Attribute name	Type	Description
1	SN	Numeric	Sample number
2	CD	Date	Catch date
3	G	String {m, f}	Gender
4	CL	Numeric	Cephalothorax length
5	CW	Numeric	Cephalothorax width
6	AL	Numeric	Abdominal length
7	AW	Numeric	Abdominal width
8	CFL-R	Numeric	Clamp foot length-right
9	CFL-L	Numeric	Clamp foot length-left
10	SL-R	Numeric	Scissor length-right
11	SL-L	Numeric	Scissor length-left
12	SW-R	Numeric	Scissor width-right
13	SW-L	Numeric	Scissor with-left
14	CH	Numeric	Cephalothorax weight
15	AH	Numeric	Abdominal weight
16	AMH	Numeric	Abdominal meat weight
17	SH-R	Numeric	Scissor weight-right
18	SH-L	Numeric	Scissor weight-left
19	SMH-R	Numeric	Scissor meat weight-right
20	SMH-L	Numeric	Scissor meat weight-left
21	TW	Numeric	Total weight
22	TL	Numeric	Total length

### Length–weight relationship and meat yield

Regression analysis is generally used to determine the relationship between body length and weight of crustaceans^22^. As in fish, there is a nonlinear relationship between length and weight in the form of Eq. (1) in crayfish. If the logarithms of both sides are taken in this equation, the length–weight relationship becomes linear as Eq. (2) ^22,23^.

1 $$W=a\times {L}^{b}$$

2 $$\text{log}W=\text{log}\left(a\right)+b \text{log}(L)$$

Abbreviations: L, total length (TL); W, total weight (TW); a and b, constant parameters of the equation.

The length–weight relationship of narrow-clawed crayfish was investigated in terms of the total length (TL)–total weight (TW) relationship. Accordingly, regression equations, curves, and correlation coefficients were calculated. While checking the significance of the calculated b value, the test statistic value was calculated using Eq. (3).

3 $$t=\frac{{S}_{x}\left|b-3\right|}{{S}_{y}\sqrt{1-{r}^{2}}}\sqrt{n-2}$$

Abbreviations: Sx, standard deviation of log (L) values; Sy, standard deviation of log (W) values); n, the number of individuals used in the calculation; r2, coefficient of determination of the log (L) and log (W) values.

To determine the meat yield, the abdomen, claws, and scissors were cut with the help of a scalpel, and the meats inside were directly weighed and their weights were determined^24^.

### Machine learning techniques

Machine learning is the general name of computer algorithms that can learn the solution to a problem, handled with complex pattern detection and data-based decision-making features^25^. Linear regression is one of the most widely used machine learning algorithms. Linear regression is a modeling method that aims to establish a linear relationship between one or more independent variables and a dependent variable or a numerical result. Therefore, this method models the relationships between dependent variables and independent variables, from analysis and learning to current educational outcomes^26^. Classification is distributing data to classes in a data set according to their attributes. Classification algorithms analyze the relationships between class labels and other features in a given training set^27^. The success of the model is determined by deciding which class the new item belongs to and testing it with the help of this model. In this study, the modeling and prediction of population growth of crayfish in Lake Apolyont were made using popular machine learning algorithms random forest regression (RFR), gradient boosting regression (GBR), decision tree regression (DTR), multilayer perceptron regression (MLPR), support vector regression (SVR), linear regression (LR) and K-nearest neighbors regression (K-NNR). Their results have been compared with each other for evaluation of achievement.

RFR is a collection of decision trees, each independent of the other and based on a random sample of training data using the same distribution. This method creates many decision trees during the training, and then, during the estimation, the classification of these decision trees is used, and the class of the input is decided by a majority vote. RF regression is discrete in that it uses multiple decision trees to produce better-fitting models and make accurate predictions. This causes it to produce the same results for the desired predictions within a between range^9,27,28^. GBR is a machine learning algorithm and a model developed to improve the prediction of decision trees. It is an algorithm developed by Friedman^29^. According to the GBR algorithm, a prediction function is first created in the first iteration and these functions are called trees. While creating the next tree, the error rates of the trees created before are kept in memory. The difference between the estimates and the observations is calculated and a loss function is obtained from these differences. In the second iteration, the difference between the predictions and the observations is calculated by combining the prediction and loss functions. Thus, it is tried to increase the success of the estimation function by adding it continuously and it is ensured that the error rate approaches zero. DTR observes the properties of an object and trains a model in the structure of a tree to predict future data to produce meaningful continuous output. Continuous output means that the output/result is not discrete, i.e., only represented by a discrete, known set of numbers or values^27,30^. MLPR is a neural network with one or more hidden layers between the input layer and the output layer. MLPR can classify nonlinear data through several hidden layers and nonlinear enable functions such as ReLU and tanh. In particular, regression analysis using MLPR does not require the assumption of a statistical relationship between independent and dependent variables. For this reason, MLPR is widely used as an algorithm for regression in various fields^31,32^. SVR is a support vector machine (SVM) implementation that generates an actual number as output. SVM can be applied to regression problems by importing an alternative loss function. SVR is built on the principle of inherent risk minimization to solve complex problems^33–35^. LR is a supervised learning algorithm that uses a parametric model and a linear approach for a prediction problem ^9,27,36^. K-NNR for machine learning is well known to have been introduced as a non-parametric approach used to classify fields and perform regressions. Within these two areas, the input data contains the closest training examples in the feature area. In K-NNR, the output is the property value of the object. This value is the average of the values of the k-nearest neighbors^36,37^. Various hyperparameters that make the machine learning methods used flexible have been tested and optimized.

Data preprocessing, which will increase the quality of raw data to be used in the study, is one of the most important processes that have a direct positive effect on computer science and the performance of all algorithms. Since machine learning algorithms are generally data-driven structures, various operations such as cleaning, scaling, reducing, and normalization have significant effects on prediction accuracy^38,39^. In this study, after the raw data were arranged and determined, they were subjected to a normalization process with min–max normalization. The applied normalization formula is given in Eq. (4).

4 $${X}_{n}=\frac{{x}_{i}-{x}_{min}}{{x}_{max}-{x}_{min}}$$

**Figure 2:**
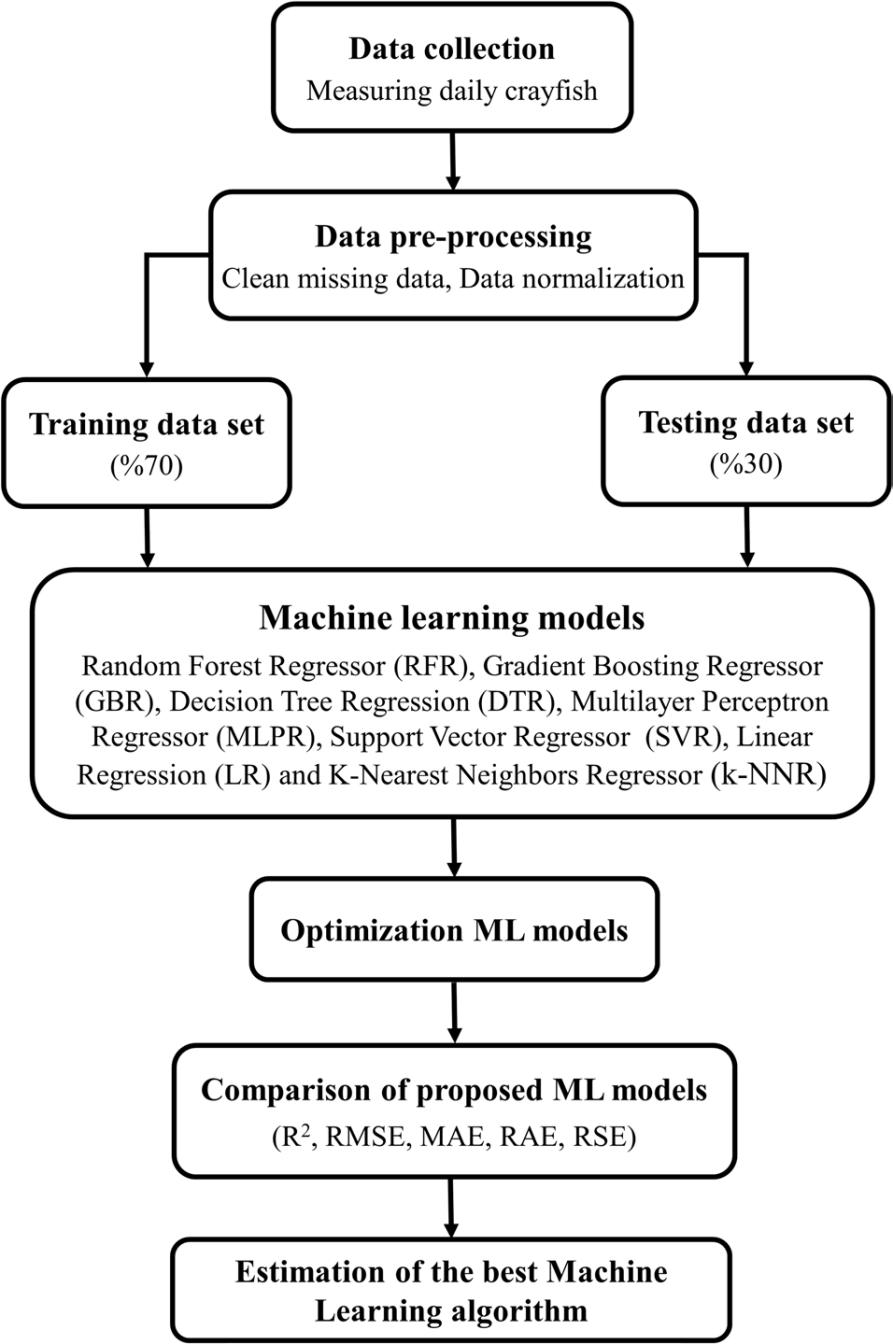
Flow diagram of the system. *R*^*2*^ coefficient of determination, *RMSE* root mean square error, *MAE* mean absolute error, *RAE* relative absolute error, *RSE* root relative square error.

In this formula, each input (x_i_) value is linearly normalized (x_n_) between 0 and 1 by finding the minimum (x_min_) and maximum (x_max_) values of the raw data set^27,39^. Of the available data, 70% was used for training the machine learning model and 30% for testing it, because the 70–30 train split helps train the model adequately on various datasets, which provides better generalization and performance evaluation^40,41^. The flow chart of the proposed method in this study is given in Fig. 2.

The models were made using a desktop on the operating system Windows 10 Pro operating system with the following hardware configuration: Intel (R) Core (TM) i7-7700HQ, 2.80 GHz processor speed, and 16.0 GB of RAM. Python language was used in the present study.

### Performance metrics

R-squared (coefficient of determination) (R^2^), root mean square error (RMSE), mean absolute error (MAE), relative absolute error (RAE), and root relative square error (RSE) values were used to measure the estimation performance of the models in the study. Using these metrics, it can be decided which technique is most suitable for this data set. In linear regression, R^2^ [Eq. (5)] is a measure of how close the data points are to the fit line. It is also known as the coefficient of determination. The R^2^ is a metric that represents prediction performance for regression models. It is a positive value between 0 and 1. The ideal value for R^2^ is 1. The closer the R^2^ value is to 1, the better the model. RMSE [Eq. (6)], prediction errors, is a measure of how far the regression line is from the data points. MAE [Eq. (7)] is the error rate of the growth forecast model. RAE [Eq. (8)] takes the total absolute error and normalizes it by dividing it by the simple estimator's total absolute error. RSE [Eq. (9)] gives the square root of the sum of the squares of the differences between the estimated value and the true value to the sum of the squares of the differences between the true values and the mean value^42^. The lower the calculated values for the four error metrics and the closer the coefficient of determination is to 1, the more accurate the results are. These five different metrics are not sufficient alone in terms of the calculated values but are meaningful when evaluated together.

5 $$R^{2} = 1 - \frac{{\mathop \sum \nolimits_{i = 1}^{n} \left( {\hat{y}_{i} - y_{i} } \right)^{2} }}{{\mathop \sum \nolimits_{i = 1}^{n} \left( {y_{i} - \overline{y}_{i} } \right)^{2} }}$$

6 $$RMSE = \sqrt {\frac{1}{N}\mathop \sum \limits_{i = 1}^{N} \left( {y_{i} - \hat{y}_{i} } \right)^{2} }$$

7 $$MAE = \frac{1}{N}\mathop \sum \limits_{i = 1}^{N} \left| {y_{i} - \hat{y}_{i} } \right|$$

8 $$RAE = \frac{{\mathop \sum \nolimits_{i = 1}^{N} \left| {y_{i} - \hat{y}_{i} } \right|}}{{\mathop \sum \nolimits_{i = 1}^{N} \left| {y_{i} - avg\left( y \right)} \right|}}$$

9 $$RSE = \sqrt {\frac{{\mathop \sum \nolimits_{i = 1}^{N} \left( {y_{i} - \hat{y}_{i} } \right)^{2} }}{{\mathop \sum \nolimits_{i = 1}^{N} \left( {y_{i} - avg\left( y \right)} \right)^{2} }}}$$

## Results

**Figure 3:**
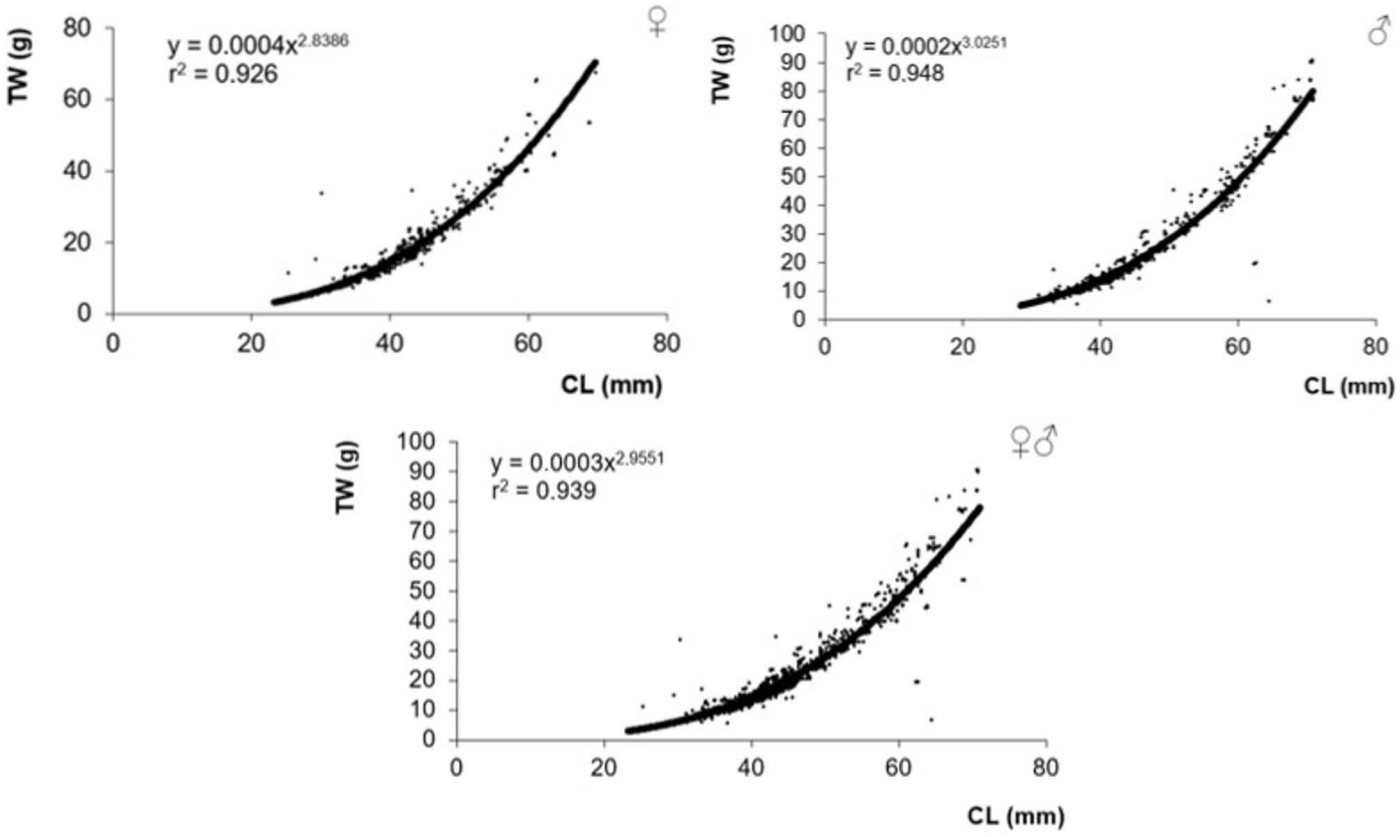
Length–weight relationship in Apolyont Lake crayfish. Symbols: ♀, female; ♂, male.

Length–weight relationships and meat yields are used to compare the characteristics of different crayfish populations. In this study, the weights of crayfish according to gender are associated with their lengths. It was seen that the crayfish ranged between 23 and 71 mm carapace lengths. The carapace length has measured a minimum of 23 maximum of 70 mm in females, a minimum of 28 maximum of 71 mm in males, and an average of 44 mm in all individuals. When the weight distributions of the crayfish were examined, it was determined that the live weight was between 2.5 and 92.4 g. The weight of female individuals ranged between 2.5 and 72.4 g, and the weight of male individuals ranged between 2.5 and 92.4 g. The average total weight measured was 20.0 g in females, and 21.9 g in males. Although there was a linear relationship between meat amounts and carapace lengths in male and female crayfish, it was determined that this relationship was stronger in male crayfish. Total meat yield was calculated as 16.45% on average in the examinations performed on a total of 1416 individuals. CL-TW relationship graphs were drawn for the entire population, including female, male, and female–male mixed (Fig. 3).

Seven different machine learning algorithms were run on the training data and the total weight performance metric results according to the length measurements of male individuals are given in Table 2 and the results of female individuals are given in Table 3.

**Table 2:** Performance metric results for total weight according to length measurement values in male crayfish.

Regression models	Performance metrics				
	R^2^	MAE	RMSE	RAE	RSE
LR	0.9365	0.0241	0.0464	0.1858	0.0635
SVR	0.9963	0.9698	0.9793	0.0851	0.0037
MLPR	0.9524	0.0205	0.0402	0.1582	0.0476
GBR	0.9878	0.0133	0.0204	0.1024	0.0122
RFR	0.9902	0.0104	0.0183	0.0798	0.0098
K-NNR	0.9704	0.0127	0.0317	0.0982	0.0296
DTR	0.9365	0.0241	0.0464	0.1858	0.064

*R*^*2*^ coefficient of determination, *RMSE* root mean square error, *MAE* mean absolute error, *RAE* relative absolute error, *RSE* root relative square error, *LR* linear regression, *SVR* support vector regression, *MLPR* multilayer perceptron regression, *GBR* gradient boosting regression, *RFR* random forest regression, *K-NNR* K-nearest neighbors regression, *DTR* decision tree regression.

**Table 3:** Performance metric results for total weight according to length measurement values in female crayfish.

Regression models	Performance metrics				
	R^2^	MAE	RMSE	RAE	RSE
LR	0.9429	0.0212	0.0295	0.2234	0.057
SVR	0.992	0.9533	0.9661	0.1144	0.008
MLPR	0.9441	0.0201	0.0291	0.2135	0.0559
GBR	0.9899	0.0094	0.0123	0.0992	0.01
RFR	0.9895	0.0088	0.0126	0.0928	0.0104
K-NNR	0.9798	0.0104	0.0175	0.1094	0.0202
DTR	0.9342	0.0198	0.0316	0.2089	0.0658

*R*^*2*^ coefficient of determination, *RMSE* root mean square error, *MAE* mean absolute error, *RAE* relative absolute error, *RSE* root relative square error, *LR* linear regression, *SVR* support vector regression, *MLPR* multilayer perceptron regression, *GBR* gradient boosting regression, *RFR* random forest regression, *K-NNR* K-nearest neighbors regression, *DTR* decision tree regression.

Likewise, the abdominal meat yield performance metric results according to the length measurements of male and female individuals are given in Tables 4, 5, respectively.

**Table 4:** Total length and abdominal meat yield efficiency of male crayfish.

Regression models	Performance metrics				
	R^2^	MAE	RMSE	RAE	RSE
LR	0.9347	0.0361	0.0486	0.2470	0.0653
SVR	0.9326	0.0386	0.0494	0.2641	0.0675
MLPR	0.9186	0.0384	0.0543	0.2631	0.0814
GBR	0.9910	0.0138	0.0180	0.0942	0.009
RFR	0.9929	0.0099	0.0161	0.0674	0.0071
K-NNR	0.9744	0.0201	0.0304	0.1375	0.0256
DTR	0.9251	0.0318	0.0521	0.2178	0.0749

R^2^ determination coefficient, *RMSE* root mean square error, *MAE* mean absolute error, *RAE* relative absolute error, *RSE* root relative square error, *LR* linear regression, *SVR* support vector regression, *MLPR* multilayer perceptron regression, *GBR* gradient boosting regression, *RFR* random forest regression, *K-NNR* K-nearest neighbors regression, *DTR* decision tree regression.

**Table 5:** Total length and abdominal meat yield efficiency of female crayfish.

Regression models	Performance metrics				
	R^2^	MAE	RMSE	RAE	RSE
LR	0.9251	0.0318	0.0521	0.2177	0.0749
SVR	0.8901	0.0467	0.055	0.3637	0.1099
MLPR	0.8878	0.0420	0.0556	0.3266	0.1122
GBR	0.9859	0.0146	0.0197	0.1135	0.0141
RFR	0.9879	0.0123	0.0183	0.0956	0.0121
K-NNR	0.9519	0.0255	0.0364	0.1985	0.0481
DTR	0.9015	0.0372	0.0521	0.2893	0.0986

R^2^ determination coefficient, *RMSE* root mean square error, *MAE* mean absolute error, *RAE* relative absolute error, *RSE* root relative square error, *LR* linear regression, *SVR* support vector regression, *MLPR* multilayer perceptron regression, *GBR* gradient boosting regression, *RFR* random forest regression, *K-NNR* K-nearest neighbors regression, *DTR* decision tree regression.

The best length–weight and length-meat yield performance metric results for all individuals were found with SVR. According to the SVR, accuracy levels were found in the length–weight prediction results of males (R^2^ = 0.996; RMSE = 0.979) and females (R^2^ = 0.992; RMSE = 0.966), and the length-meat yield prediction results of males (R^2^ = 0.996; RMSE = 0.098) and females (R^2^ = 0.995; RMSE = 0.977) were 99.6%, 99.2%, 99.6%, and 99.5%, respectively. The lowest accuracy levels were found with MLPR for female length–weight, male and female length-meat yield performance metric results. On the other hand, in male length–weight performance metric results, the lowest accuracy level was found with DTR.

## Discussion

Knowing the population’s current status is important in determining the fishing strategies in fisheries management. The basic idea is to make estimates of population size at regular intervals. This is a tiring job that requires money and effort; however, the change in the population is observed by doing it at least every few years. Because population size depends on time in a certain period, death, reproduction, migration, growth, catching, abundance of food, predators, etc. changes in number and weight with the effect of factors^43,44^. Stock monitoring studies that need to be carried out uninterruptedly for the sustainability of populations and the determination of growth characteristics in population parameters are important for the evaluation of the population. Length differences between body parts are used to show morphological changes between male and female individuals of crayfish species. These differences are also used to determine the relative growth of crayfish populations and to compare populations of the same species. The meat yield of individuals in the population is an important parameter used in population estimation under extensive conditions. The exact determination of the population size is possible by catching all individuals. Since it is impossible to do this in practice, the population size is determined with the help of the population parameters explained here. Therefore, it was aimed to determine these growth characteristics of crayfish in this study.

Machine learning provides a neutral approach to recognizing unknown interactions and deriving predictions that have the potential to aid in meaningful feature selection. The correlation values produced as a result of the algorithm estimates show the correlation between length–weight and length-meat yield measurement values and length–weight and length-meat yield prediction values. Since this study is based on the properties that are effective in the correct estimation of the length–weight and length-meat yield values with the determination of the correct length–weight and length-meat yield prediction, they are evaluated on the positive correlation values formed as a result of the estimates of the algorithms. Because the proximity of correlation values to + 1 indicates the closeness of length–weight and length-meat yield estimates to length–weight and length-meat yield measurements.

In the present study, SVR performance metric results are 0.996 and 0.992 values both for the length–weight of male and female individuals, with 0.996 and 0.995 values and for the length-meat yield of males and females are closer to the values of measurement. SVR stands out as the method with the best R^2^ and error values. SVR is both linear and non-linear^35^ due to this feature, it is considered that the SVR provides better performance in evaluating the population structure. Similarly, Benzer et al.^8^, and Benzer and Benzer^45^, showed that ANN could be a superior estimation tool compared to the length–weight for the growth predictions of narrow-clawed crayfish in Hirfanlı Dam Lake and Uluabat Lake, respectively. In addition, SVM gave 80% accuracy in classifying sea bream feeding trials according to hematological blood parameters. In addition, SVM provided 80% accuracy in classifying sea bream (*Sparus aurata*) feeding trials according to hematological blood parameters^9^, but K-NN gave better results than SVM in *Alburnus tarichi* population analysis^35^. All these studies showed that artificial intelligence applications such as ANN and machine learning etc. should be specific to data. Before evaluating a population in the long term, the first thing to do is to determine which application is appropriate for that population.

**Figure 4:**
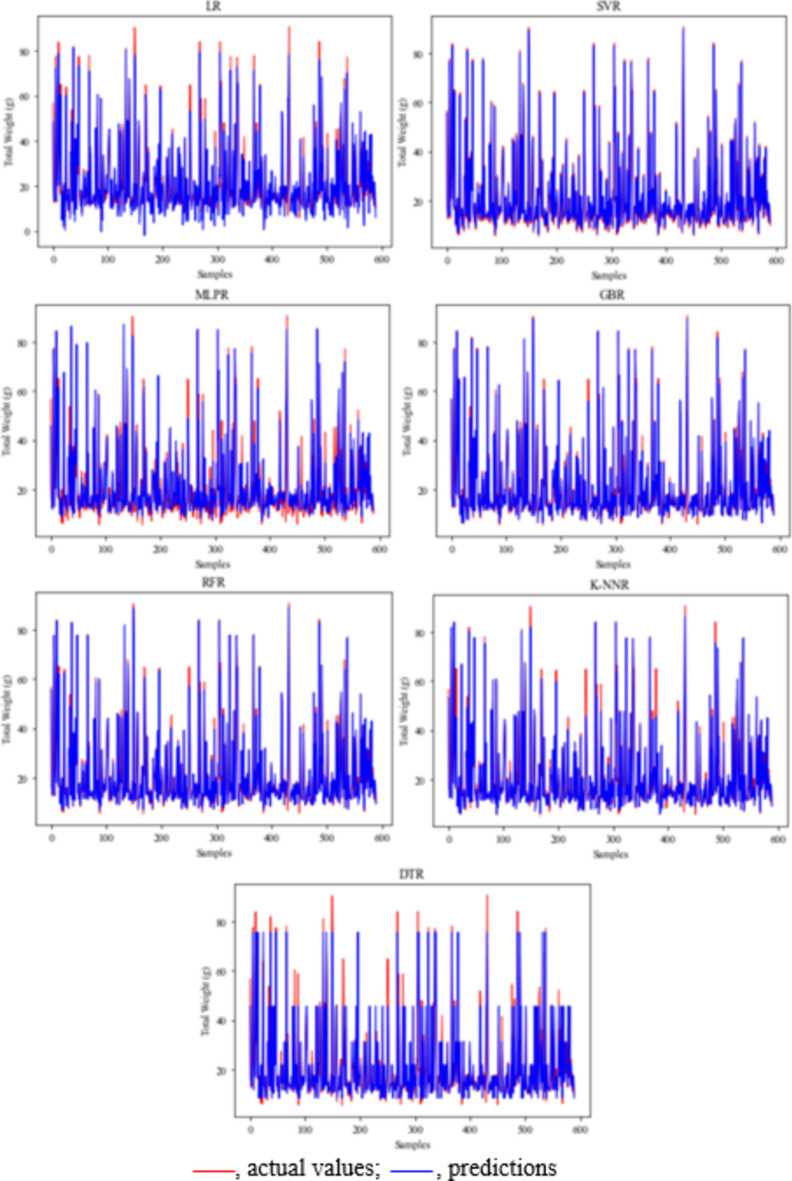
Length–weight prediction and actual values in male crayfish. *LR* linear regression, *SVR* support vector regression, *MLPR* multilayer perceptron regression, *GBR* gradient boosting regression, *RFR* random forest regression, *K-NNR* K-nearest neighbors regression, *DTR* decision tree regression.

**Figure 5:**
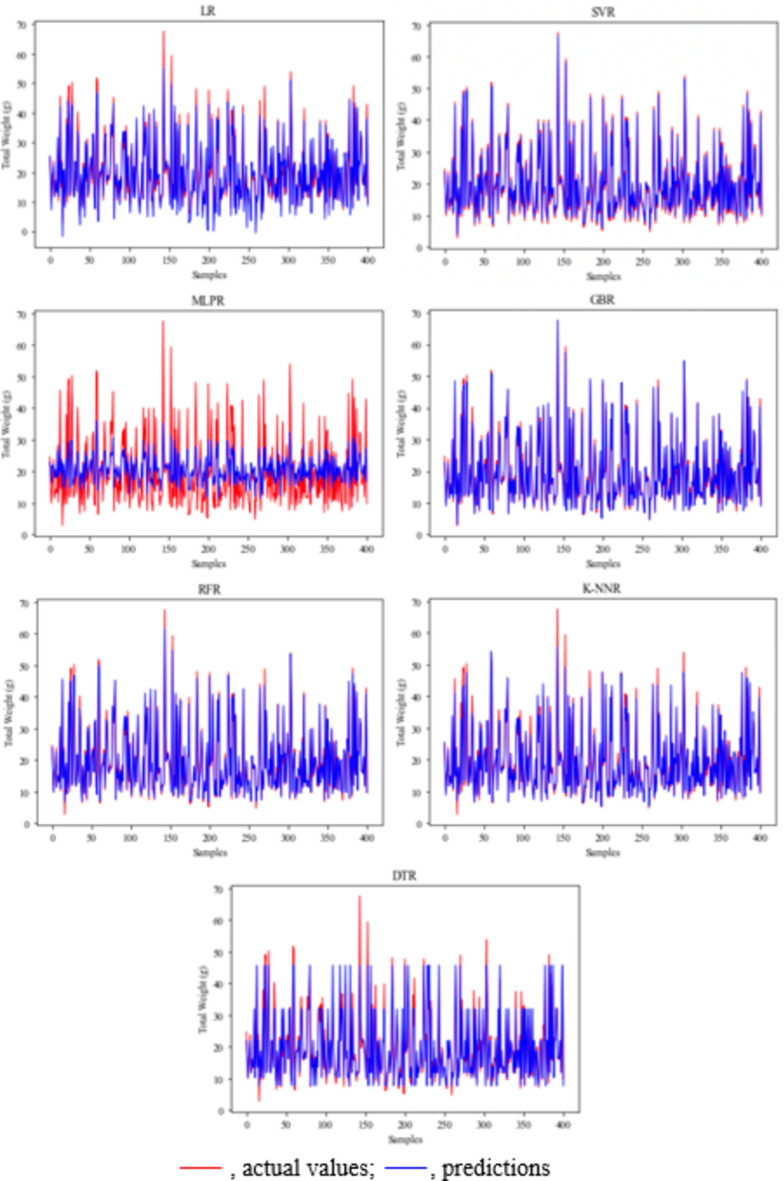
Length–weight prediction and actual values in female crayfish. *LR* linear regression, *SVR* support vector regression, *MLPR* multilayer perceptron regression, *GBR* gradient boosting regression, *RFR* random forest regression, *K-NNR* K-nearest neighbors regression, *DTR* decision tree regression.

**Figure 6:**
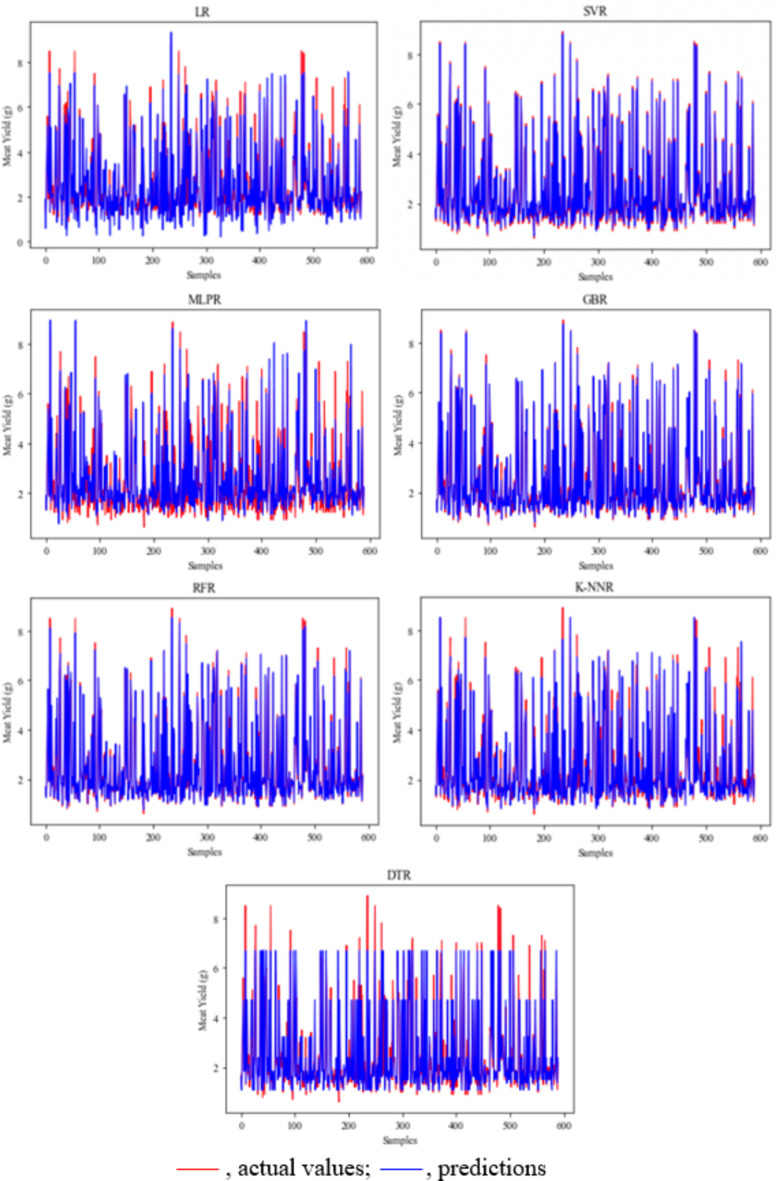
Meat yield prediction and actual values in male crayfish. *LR* linear regression, *SVR* support vector regression, *MLPR* multilayer perceptron regression, *GBR* gradient boosting regression, *RFR* random forest regression, *K-NNR* K-nearest neighbors regression, *DTR* decision tree regression.

**Figure 7:**
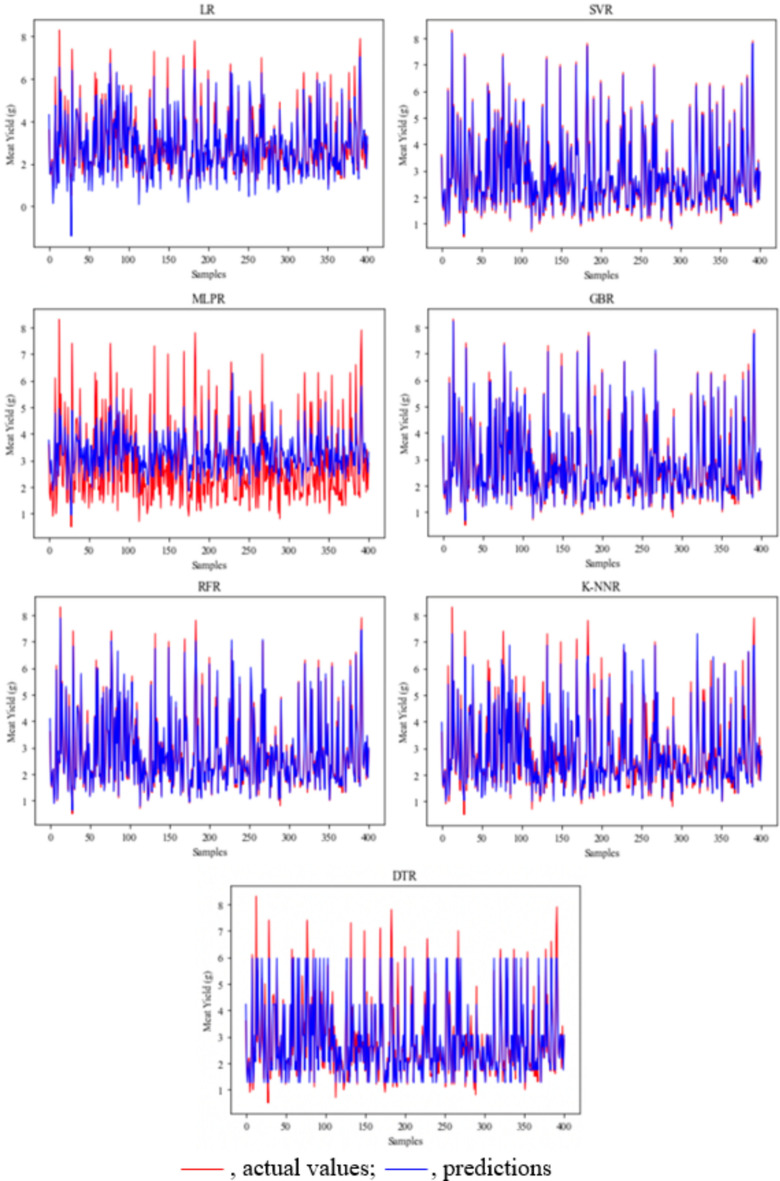
Meat yield prediction and actual values in female crayfish. *LR* linear regression, *SVR* support vector regression, *MLPR* multilayer perceptron regression, *GBR* gradient boosting regression, *RFR* random forest regression, *K-NNR* K-nearest neighbors regression, *DTR* decision tree regression).

The results of this study showed that SVR is the most successful application to evaluate the crayfish population living in Apolyont Lake. To better evaluate the results, the prediction and actual values were given for male length–weight in Fig. 4, for female length–weight in Fig. 5, for male length-meat yield in Fig. 6, and for female length-meat yield in Fig. 7.

Ecological factors determining the presence of white-clawed crayfish (*Austropotamobius pallipes*) were evaluated using SVM, a machine-learning method. It was determined that the models without feature selection, the models created by applying Goldberg’s genetic algorithm after the feature selection, and the models built after selecting inputs using the four supervised-filter evaluators had a classification accuracy degree of 70.84%, 73.92%, 76.62%, respectively^16^. The applied model in this study has higher accuracy as well as found closer to the real values. Thus, the study results of Zelaya^38^ were supported and the accuracy of the results obtained from this study was observed. Besides, Tirelli et al.^7^ used logistic regression, decision tree models, and artificial neural networks to manage data on the presence/absence of native white-clawed crayfish. They obtained better performance from logistic regression and decision tree models using the artificial neural network model. However, a hybrid three-dimensional (3D) dissolved oxygen content estimation model based on a radial basis function neural network, K-means, and subtractive clustering effectively demonstrated the three-dimensional distribution in predicting changes in dissolved oxygen content on crap pools^46^. Many crayfish cannot be sold at the end of the sales period due to errors made in production planning, growth estimation, transportation, grading processes, and the application of the appropriate stock density. This situation causes a loss of resources, energy, and capital in both the production and sales stages. In the present study, it was seen that due to the multivariate nature of predicting the growth rate of crayfish populations, instead of creating hybrid models, it can be done by using machine learning methods that give faster and more accurate results in complex structured data sets.

## Conclusion

This is the first study to use machine learning methods to predict the total length-total weight and total length-meat yield performance of the narrow-clawed crayfish population of Apolyont Lake. The results show that machine learning methods can predict population growth rates and size in fisheries and aquatic populations. Among these seven machine learning applications implemented, SVR gave the best performance in terms of both R^2^ value and error metrics. In the study, it was seen that the values obtained by machine learning have high performance and are closer to the real values. The experimental results of the study show that the proposed method can be used as an effective population measurement estimation tool. Moreover, this article, which presents a series of empirical analyses with the results discussed, is considered to be valuable for professionals of natural resource management, fisheries, aquaculture, and their sustainability.

### Supplementary Information

Supplementary Information.

## Data Availability

All data that support the findings of this study are included within this paper and its Supplementary Information files.
